# Effect of Searing Process on Quality Characteristics and Storage Stability of Sous-Vide Cooked Pork Patties

**DOI:** 10.3390/foods9081011

**Published:** 2020-07-27

**Authors:** Dong Kook Cho, Boin Lee, Hyeonbin Oh, Jae Sang Lee, Young Soon Kim, Young Min Choi

**Affiliations:** 1 Department of Integrated Biomedical and Life Sciences, Korea University, Seoul 02841, Korea; chocookhouse@hanmail.net (D.K.C.); irnark@naver.com (H.O.); 2 Department of Animal Science, Kyungpook National University, Sangju-si 37224, Korea; ananassab@knu.ac.kr; 3 Department of Hotel Culinary, Kyungdong University, Yangju 11458, Korea; leecook@kduniv.ac.kr

**Keywords:** sous-vide, searing, quality traits, sensory quality, storage stability, pork patties

## Abstract

This study investigated the effects of searing process before sous-vide (SV) treatment on quality traits, visual attributes, palatability, and storage stability of SV cooked pork patties. Patties were seared on each side by pan-frying for 0 (control), 30 (S30), 60 (S60), 90 (S90), or 120 (S120) s in a stainless-steel pan, and all patties were then vacuum-packed and cooked under thermally controlled conditions at 75 °C for 2 h. Marked differences were observed in quality properties between the control and searing groups, and the S120 group exhibited greater brown surface color and cooking loss compared to the other groups (*p* < 0.001) due to the additional heating process. Patties from the S60 group showed greater appearance and tenderness acceptability scores compared to patties from the S30 and S120 groups (*p* < 0.001). On another note, the effects of searing on storage stability were somewhat limited, as they were measured by 2-thiobarbituric acid reactive substance, volatile basic nitrogen, total aerobic bacterial count, and coliforms during 49 d of cold storage. Therefore, searing process before SV treatment can improve the visual attributes and palatability of cooked pork patties, and the optimum searing condition was for 60 s, without impairing the storage stability.

## 1. Introduction

Consumer habits are changing along with economic and technological developments, and consumers are now demanding simpler, cleaner, healthier, and more convenient food products [1]. Due to these consumer demands, the food industry has been increasing the food products with minimally processing and extended shelf-life, especially ready-to-eat (RTE) foods [2]. Sous-vide (SV) is a well-known method fulfilling consumer demands for RTE meat-based foods and is defined as cooking under preciously controlled low temperature long time (LTLT) heat conditions in vacuum-sealed food-grade plastic pouches [3,4]. SV cooking conditions, including vacuum-sealing, can increase shelf-life by eliminating contamination risks and inhibiting off-flavors from lipid oxidation [3,5]. Additionally, the precisely controlled temperature during SV cooking allows uniform and improved palatability, especially tenderness, by efficiently and evenly transferring heat to meat products compared to traditional cooking methods [4,6]. Due to these benefits, SV cooking was originally developed for the catering industry and often used by high-end restaurant chefs as well as consumers at home for the various meat products, such as steaks, sausages, and patties [7,8].

On the other hand, although desirable tenderness and juiciness are obtained through the SV cooking method, it is possible to reduce the appearance acceptability of consumer since there is lack of brown surface color in SV cooked meat [9]. Moreover, SV cooked meat products generally show less flavor in comparison with products cooked at a high temperature, as flavor compounds are less formed at LTLT conditions due to the lack of extensive Maillard reactions [6]. Thus, additional treatment is needed to overcome these drawbacks, and searing before or after LTLT cooking is one of the common methods to attain a desirable appearance and flavor of loin steaks [6,9]. However, there is limited information about the effects of searing process on appearance acceptability and palatability of SV cooked patties. Therefore, the aim of this study was to investigate the effects of searing process on quality traits, visual attributes, sensory quality traits, and storage stability of SV cooked pork patties.

## 2. Materials and Methods

### 2.1. Pork Patty Production and Treatments

A total of 48 kg (12 kg per batch) of fresh pork ham (*biceps femoris*, *semitendinosus*, *gracilis*, *semimembranosus*, *adductor*, and *pectineus* muscles separated from 4 carcasses) and back fat were purchased from a livestock vendor (Korea Prime Meat Industry Co., Seoul, Korea). The basic formulation of pork patty was 80% lean meat, 18.5% back fat, and 1.5% salt. After trimming the visible fats and connective tissues, pork ham and fat were ground using a meat grinder with a 3 mm plate (Twin 275/114, Kutter-und Gerätebau Wetter GmbH, Biedenkopf, Germany), and mixed together for 3 min in a mixer (HL200, Hobart, OH, USA). Sterilized petri dishes (7 cm diameter and 1.5 cm height) were used to produce patties with constant size and weight (60 ± 1.5 g each), and a total of 190 patties per batch were wrapped with polyethylene film. Pork patties were immediately frozen at −18 °C, and 12 frozen patties were then placed and vacuum-packed in each polyethylene pouch (250 × 350 mm; O_2_ permeability of 9 cm^3^/m^2^ per 24 h at 4 °C and water steam permeability of 1.2 g/m^2^ per 24 h at room temperature) using a vacuum packaging machine (Leepack, Hanguk Electronic, Kyunggi-do, Korea), and stored at −18 °C until further experiments. The pork patty production was repeated four times. Frozen pork patties were thawed at 4 °C overnight, and 38 samples per group per batch (total 5 groups; 38 samples × 5 groups = 190 patties) were randomly selected. Samples were then seared on each side by pan-frying for 0 (control), 30 (S30), 60 (S60), 90 (S90), or 120 (S120) sec in a preheated stainless-steel pan (28 cm diameter) at 180 °C using an electric induction range (CIR-IH300RGL, Cuchen, Seoul, Korea) set to the 5th heating level. All patties from control and searing groups were then consecutively vacuum-sealed in a nylon-polyethylene pouch (150 × 200 mm) using a vacuum packaging machine (Chamber Vacuum Sealer System 300 Series, Poly Science Innovation Culinary Technology, Denver, CO, USA) with a 98.81% vacuum degree. For the SV cooking treatment, the patty samples were cooked using a circulating temperature-controlled sous-vide machine (Sous-vide cooker, Fusionchef by Julabo, Seelbach, Germany), and cooking conditions were at 75 °C for 2 h. All SV cooked patties were cooled in an ice-slurry until equilibration; then, the quality characteristics and cooking properties were immediately examined using 10 patties per group; another 10 patties were stored at −18 °C for visual attributes and sensory quality trait assessment, and the remaining 18 samples (3 patties per storage day) were stored at 4 °C for storage stability measurement.

### 2.2. Quality Measurements and Cooking Properties

SV cooked patties were used for pH measurement using a portable pH instrument (Testo 206-pH2, Testo AG, Lenzkirch, Germany). Surface color values, including L*, a*, and b*, were measured using a Minolta colorimeter (CR-400, Minolta Camera Co., Osaka, Japan) according to the recommendations of the Commission Internationale de l’Eclairage [10], and hue angle [tan^−1^(b*/a*)] and saturation index [(b*^2^ + a*^2^)^0.5^] were calculated. The colorimeter parameters were consistent with those previously reported by Lee et al. [11]. For texture profile analysis (TPA), more than ten cubes (20 × 20 × 15 mm) were used, and a double compression cycle test was performed. Analysis was conducted using a TMS-touch texture analyzer (Food Technology Co., Sterling, VA, USA) with the crosshead speed set to 180 mm/min, compression to 70% of the original height, and max load cell of 10 kg. Hardness, cohesiveness, springiness, and chewiness were determined using the method previously described by Bourne [12].

Cooking yields were calculated using standard procedures as previously reported by Choi et al. [13]. Diameter and thickness of SV cooked samples were determined using a stainless-steel Vernier caliper (ABSOLUTE Digimatic caliper, Mitutoyo Co., Kawasaki, Japan), and the percentage of diameter, thickness, and shrinkage changes were calculated [13,14].

### 2.3. Visual Attributes and Sensory Quality Characteristics

For visual attributes and palatability analyses, a total of 200 pork patties were used during 12 sessions (3–4 patties × 5 groups per session). Before each session of the sensory evaluation, frozen patty samples were thawed at 4 °C for 5 h, and then thawed patties were heated to an internal temperature of 54 °C in a water bath [13]. The core temperature of sous-vide patties was maintained in a water bath until served to trained panelists. Ten panelists (five women and five men; 24 to 45 years old) were trained according to the procedures of American Meat Science Association [15] and Meilgaard et al. [16], and these panelists evaluated the visual attributes and sensory quality traits of the SV cooked patties. Panelists were selected from researchers and faculty of Kyungpook National University (KNU), and underwent further training of sensory quality characteristics of meat and meat products at least 1 year. All training and sensory evaluation sessions were conducted at KNU, and human ethics approval was granted by the Bioethics Committee of KNU (protocol number: 2019-0027). During sensory quality evaluations, trained panelists were served unsalted crackers and sufficient water prior to the first patty sample and between patty samples for refreshment of panelist plates. The trained panelists evaluated the visual attributes, including color (very pale–very dark), moisture (very dry–very moisture), appearance acceptability (very unacceptable–vey acceptable), and overall acceptability (very unacceptable–vey acceptable) using a 9-point hedonic scale. Sensory quality of cooked pork patties was then evaluated for 6 attributes, including tenderness acceptability (1–9; very unacceptable–very acceptable), juiciness (1–9; extremely dry–extremely juicy), flavor intensity (1–9; no patty flavor–full flavor), off-flavor intensity (1–9; very strong–very weak), saltiness (1–9; very weak–very strong), and overall acceptability (1–9; very unacceptable–very acceptable).

### 2.4. Storage Stability

To assess the storage stability of SV cooked patties during storage periods from 0 to 49 d, values of 2-thiobarbituric acid reactive substance (TBARS), volatile basic nitrogen (VBN), total aerobic bacteria, and coliforms were measured. The lipid oxidation extent was determined using the TBARS content in SV cooked patties during cold storage periods (0, 7, 14, 21, 35, and 49 d) at 4 °C by method previously described by Buege and Aust [17]. A total of three repetitions were conducted, and the values were expressed as milligrams of malondiadehyde (MDA) per kg of patty sample. VBN concentrations were measured following the method described by Conway [18], with slight modifications. Each 10 g patty was homogenized with 90 mL of distilled water using a homogenizer (Unidrive1000D, CAT M. Zipperer GmbH, Staufen, Germany). Homogenized sample was filtered using a Whatman No. 1 filter paper (Whatman, Maidstone, UK). Then, 1 mL of the sample was added to the outer-chamber of a Conway micro-diffusion cell, and 0.01 N H_3_BO_3_ was add to the inner cell, whereas 1 mL K_2_CO_3_ was added to the outer cell. The cell was horizontally shaken and incubated at 37 °C for 2 h. The mixture was titrated with 0.02 N H_2_SO_4_. The experiment was conducted in triplicate, and VBN content was calculated using a method previously described by Kim et al. [19]. Then, 10 g of SV cooked patty was homogenized with 90 mL sterilized saline solution using a homogenizer (Pulsifier PUL100E, Microgen Bioproducts Ltd., Surrey, UK). After diluting the homogenate, total aerobic bacteria count and coliform counts were determined using 3M petrifilm (3M Company, St. Paul, MN, USA) after incubation at 37 °C for 48 h. Results were expressed as log colony forming unit (CFU)/g.

### 2.5. Statistical Analysis

The general linear model (GLM) procedure (SAS software, SAS Institute, Cary, NC, USA) was used to compare quality traits, cooking properties, sensory quality characteristics, and storage stability among the treatments. Regarding the quality characteristics and cooking properties, a linear mixed model was used to identify the factors influencing the response. The model included searing treatments as fixed effect and repetitions as random effect. Regarding the visual and sensory quality characteristics of sous-vide cooked patties, a linear mixed model was used to analyze these quality attributes of each sample to identify the factors influencing the response. The model included searing treatment as fixed effect, and panelists and repetitions (4 repetitions; 10 samples from each group per each repetition) as random effects. For the values of TBARS and VBN, the model included searing treatments and storage periods as fixed effects and repetitions as random effect. Significant differences were compared by the probability difference (PDIFF) option at *p* < 0.05. Data were presented as the means of least-squares and standard errors.

## 3. Results

### 3.1. Effects of Searing on Quality and Cooking Properties of Sous-Vide Patties

Table 1 presents the effects of searing process on the quality traits of SV cooked pork patties. No difference was observed in pH between the searing treatments (*p* > 0.05), although the control group showed a lower pH value compared to the S30 and S120 groups (6.20 vs. 6.23 and 6.25, *p* < 0.001). The control group showed a higher lightness value (55.8 vs. 46.8, *p* < 0.001) and lower redness (5.08 vs. 6.48, *p* < 0.001) and yellowness (9.21 vs. 12.0, *p* < 0.001) values compared to the S120 group. Lightness value was similar between the control and S30 groups (*p* > 0.05), but significant differences were observed in redness and yellowness values between the two groups (*p* < 0.001). The S30 and S60 groups exhibited higher values of hue angle compared to the control group (70.3 and 70.1 vs. 61.2, *p* < 0.001), and saturation index was lower in the control group compared to the searing groups (*p* < 0.001). All seared SV pork patties exhibited a higher value of TPA-hardness compared to control SV patties (*p* < 0.001). The S30 group showed a higher value of cohesiveness compared to the control group (0.70 vs. 0.64, *p* < 0.001), and a lower value was observed in the S120 group compared to the S60 group (0.47 vs. 0.65, *p* < 0.001). Higher springiness (1.73 vs. 1.33 mm, *p* < 0.001) and chewiness (5.24 vs. 3.50 N·mm, *p* < 0.001) values were detected in the S90 group compared to the control group.

As expected, the searing groups significantly differed in terms of cooking properties (Table 2). Cooking yield was significantly decreased with increasing searing process time. Sous-vide patties from the control group showed a higher value of cooking yield compared to sous-vide patties from the searing groups (*p* < 0.001), and the S120 group exhibited the lowest yield (73.4%, *p* < 0.001). The treatments showed significantly higher changes in diameter (*p* < 0.001) and shrinkage (*p* < 0.001) compared to the control group. S120 patties had a higher change in thickness than control patties (41.1 vs. 34.5%, *p* < 0.001), although no difference was observed between the control and S90 groups (*p* > 0.05).

### 3.2. Effects of Searing on Sensory Quality Characteristics of Sous-Vide Patties

The effects of searing process on the visual attributes of SV cooked patties are presented in Table 3. Marked difference was observed in color intensity among the groups (*p* < 0.001), although there was no difference between the S90 and S120 groups (7.17 vs. 7.17, *p* > 0.05). The control and S30 patties (6.11 and 5.70, respectively) did exhibit a higher moisture intensity than the S90 and S120 groups (4.88 and 4.29, *p* < 0.001), although no significant difference was observed between the control and S30 groups (5.70, *p* > 0.05). However, sous-vide pork patties from the control group exhibited lower appearance and overall acceptability compared to sous-vide pork patties from the searing groups (*p* < 0.001). There were no differences in appearance (5.41 vs. 5.41, *p* > 0.05) and overall acceptability (5.42 vs. 5.78, *p* > 0.05) between the S30 and S120 groups.

Table 4 displays the sensory quality characteristics for the control and searing groups. SV cooked patties with searing showed a higher value of tenderness acceptability compared to the SV patties without searing (*p* < 0.001) with the exception of the S120 group (*p* > 0.05). There was no significant difference in tenderness acceptability between the S120 and S30 groups (*p* > 0.05). The control groups showed a higher juiciness score compared to the S90 group (5.73 vs. 4.55, *p* < 0.001). Flavor intensity tended to increase along with searing time (*p* < 0.001), although no difference was observed between the S90 and S120 (7.16 vs. 7.28, *p* > 0.05) groups. No difference was detected in off-flavor intensity among the groups (*p* > 0.05). On the other hand, saltiness tended to decrease with increasing searing process time (*p* < 0.001), although there was no difference between the control and S30 groups (5.73 vs. 5.44, *p* > 0.05). The searing groups, with the exception of the S120 group, showed a higher score for overall acceptability compared to the control group (*p* < 0.001).

### 3.3. Effects of Searing on Storage Stability of Sous-Vide Patties

**Figure 1:**
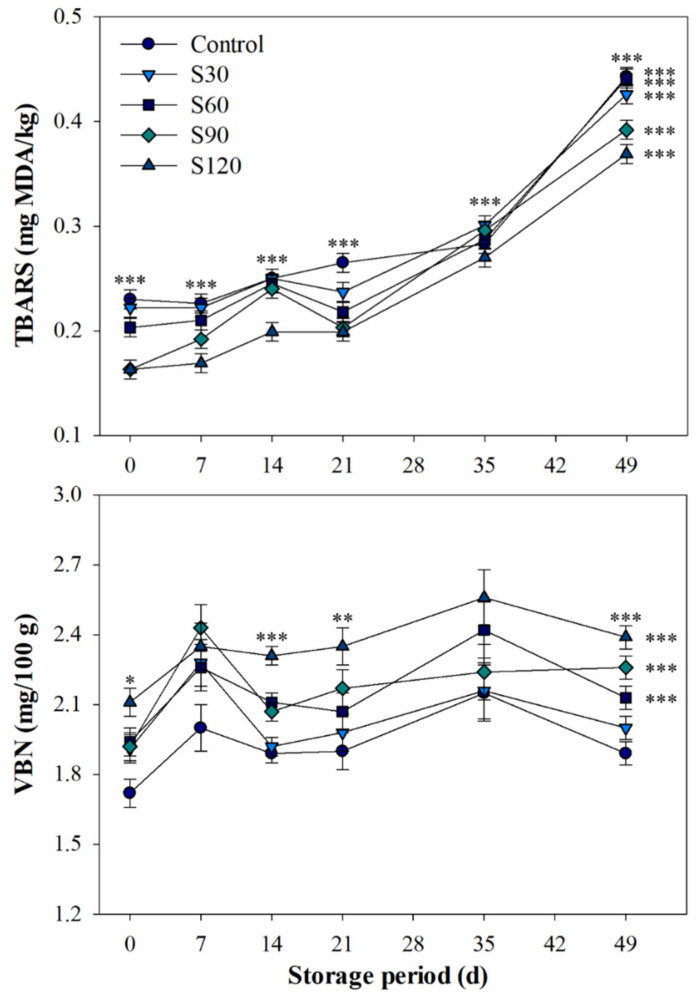
Effects of searing process on 2-thiobarbituric acid reactive substance (TBARS) and volatile basic nitrogen (VBN) values of sous-vide cooked pork patties during storage. Bars indicate standard errors of means. Level of significance: * *p* < 0.05; ** *p* < 0.01; *** *p* < 0.001.

Changes in TBARS and VBN concentrations among the groups during storage periods are presented in Figure 1. All SV cooked patties tended to increase TBARS values during 0–49 d of cold storage. After 14 d of storage, all patties within each group exhibited a higher TBARS value compared to that at 0 d of storage (*p* < 0.001), and also showed a higher value at 49 d of storage compared to 14 d of storage (*p* < 0.001). The control group showed higher concentrations at 0 and 49 d compared to the searing groups (*p* < 0.001), and the S120 group did exhibit the lowest values at 7, 14, 35, and 49 d of storage compared to the other groups (*p* < 0.001). No difference was observed in the VBN concentrations during storage periods within the control or S30 groups (*p* > 0.05). SV patties from the S90 (1.92 vs. 2.26 mg/100 g, *p* < 0.001) and S120 (2.11 vs. 2.39 mg/100 g, *p* < 0.001) groups showed higher values of VBN at 0 d compared to at 49 d. On the other hand, there was no difference between the groups at 7 and 35 d of storage (*p* > 0.05). Lower concentration was detected in the control group at 0 d compared to the searing groups (*p* < 0.05); whereas, no difference was observed at 49 d between the control and S30 groups (1.89 vs. 2.00 mg/100 g, *p* > 0.05).

The effects of searing process on the microorganisms in SV cooked patties are presented in Table 5. After searing process, a marked difference was observed in the total aerobic bacterial counts between the control and searing groups (*p* < 0.001), and control patties did exhibit a higher bacterial count compared to the S30 group (8.43 vs. 1.88 log CFU/g). After SV cooking, aerobic bacteria were not detected in all patties during storage periods from 0 to 49 d. Coliforms in pork patties tended to decrease with increasing searing process time (*p* < 0.001), although no difference was observed between the control and S30 groups after searing (0.76 vs. 0.83 log CFU/g, *p* > 0.05). Similar to the total aerobic bacterial counts, coliforms were not detected in all patties after SV cooking during cold storage.

## 4. Discussion

SV cooked food products are mainly used in catering service and restaurants, and SV processing has been applied to meat products at various cooking temperature (50–100 °C) and time (more than 1 h) conditions [1,5,20]. The recommended SV temperature and time conditions for beef and lamb steaks to enhance quality characteristics and storage stability are at 58–63 °C for long time (10–48 h) [3] and between 75–80 °C for pork [8]. Park et al. [4] recommended that the optimum cooking conditions for chicken breasts are at 60 °C for 2 or 3 h to improve tenderness attributes without impairing the cooking yield. In the current study, a combination of temperature (75 °C) and time (2 h) conditions was implemented for the SV cooking method, and searing process was additionally performed to improve the quality traits of pork patties. Due to additional heating, the seared SV patties searing exhibited a darker surface compared to the unseared SV patties, although no difference was observed between the control and S30 patties (*p* > 0.05). Hue angle value, which represents the shift in patty color from red to yellow, was higher in the searing groups, with the exception of the S120 group, compared to the control group (*p* < 0.001) due to a higher redness value. For saturation index, expressed as brightness of color, the unseared patties presented less vividness compared to the seared patties (*p* < 0.001). TPA-hardness tended to increase along with searing time (*p* < 0.001); however, the hardness value of the S60 group was similar to the S90 group (*p* > 0.05). The textural characteristics of cooked patties are generally associated with cooking properties, especially treatment loss [13,21]. During cooking, these cooking properties are influenced by the extent of protein denaturation, loss of melted fat and juices, and water evaporation [21]. In this study, significant differences were detected in cooking yield and shrinkage percentages depending on the searing time, and the cooked patties from the S60 group showed higher changes in cooking loss and shrinkage compared to the patties from the control group (*p* < 0.05).

Searing process causes meat surface dehydration, which increases the Maillard reaction development [9,22]. The extent of Maillard reaction development affects the brownness of the meat surface, and consumers generally prefer brown-surfaced steaks or patties rather than those with paler surface [3]. Thus, steaks and meat patties commonly require searing or saucing [9,23]. These results are associated with the visual attribute results of seared SV patties in this study, and a higher score of color intensity and a lower score of moisture intensity were observed in the S120 group compared to the S30 and S60 groups (*p* < 0.001). Patties from the S120 group exhibited lower appearance and overall acceptability due to excessive dehydration and their relatively brown surface compared to patties from the other searing groups (*p* < 0.001) with the exception of the S30 group. The trained panelists preferred the appearance of patties from the S60 group rather than the control and other searing groups (*p* < 0.001). Regarding eating quality characteristics, SV pork patties from the S60 and S90 groups were rated with acceptable tenderness compared to patties from the S120 group (*p* < 0.05), although a firmer structure was observed in terms of TPA-hardness in the S120 group compared to in the other groups (*p* < 0.001). On the other hand, the SV patties with longer searing time, especially the S120 group, had a relatively higher flavor score compared to the SV patties with shorter searing times or without searing (*p* < 0.001), as various flavor compounds are enhanced by cooking conditions, including time and temperature [2,9,24]. However, due to higher water loss in patties with longer searing time, the panelists distinguished a difference in saltiness between the S120 and S60 groups (*p* < 0.001), and no difference was observed between the control and S60 groups (*p* > 0.05). Due to these results, the S60 group exhibited a higher score in the overall acceptability compared to the other searing and control groups. Generally, vacuum packing and the precise temperature control used in the SV treatment of meat products can inhibit lipid oxidation and aerobic bacterial growth during storage, which improve storage stability [3,25]. TBARS concentration is generally used as an indicator of lipid oxidation extent in meat products, which is positively correlated with off-flavor intensity [19,26]. In the current study, SV pork patties with searing tended to exhibit lower TBARS contents compared to patties without searing at 0 and 49 d of cold storage (*p* < 0.05). Especially, patties from the S90 and S120 groups exhibited lower TBARS contents compared to patties from the control and S30 groups (*p* < 0.05). These results can be explained by the previous results from Benjakul et al. [27] and Morales and Jimenz-Perez [28], who suggested that various compounds derived from the Maillard reaction have strong antioxidant activity, and thus, Maillard reaction products have been used for the prevention of lipid oxidation including meat products. However, all SV patties in this study did not have TBARS values that consumers would perceive as having an off-flavor, since the threshold for TBAR values in meat products is above 0.5 mg MDA/kg [29]. Additionally, according to the Food Code in Korea [30], the VBN concentration of meat products is based on 20 mg/100 g, a value above which meat is considered as spoiled. Thus, all patties in this study were considered to maintain storage stability at 49 d of storage period, although the seared patties exhibited a higher VBN value at 0 d compared to the unseared patties (*p* < 0.001). During cold storage from 0 to 49 d, aerobic bacteria and coliforms were not detected in all cooked patties in this study. Therefore, the searing process did not significantly affect the storage stability of SV cooked patties.

## 5. Conclusions

Searing process before sous-vide treatment can improve the appearance and sensory quality acceptability, although a higher treatment loss was observed in seared pork patties compared to that in unseared patties. Patties with longer searing time exhibited poor cooking properties and surface color than patties with shorter searing time, especially the S60 group. Overall, the optimum time condition for searing process of sous-vide cooked pork patties was for 60 s in order to improve palatability without impairing the storage stability.

## Figures and Tables

**Table 1:** Effects of searing process on quality characteristics of sous-vide cooked pork patties.

	Control	Searing Treatment				SEM	Level of Significance ^1^
		S30	S60	S90	S120		
pH	6.20 ^b^	6.23 ^a^	6.24 ^a^	6.24 ^a^	6.25 ^a^	0.01	***
Lightness (L^*^)	55.8 ^a^	54.3 ^ab^	53.9 ^b^	48.8 ^c^	46.8 ^d^	0.56	***
Redness (a^*^)	5.08 ^b^	3.85 ^c^	4.73 ^b^	4.98 ^b^	6.48 ^a^	0.16	***
Yellowness (b^*^)	9.21 ^d^	10.8 ^c^	13.0 ^a^	11.7 ^b^	12.0 ^b^	0.18	***
Hue angle ^2^	61.2 ^c^	70.3 ^a^	70.1 ^a^	66.8 ^b^	61.7 ^c^	0.69	***
Saturation index ^3^	10.5 ^d^	11.4 ^c^	13.9 ^a^	12.7 ^b^	13.6 ^a^	0.20	***
*Texture profile analysis*							
Hardness (N)	4.15 ^d^	5.36 ^c^	5.85 ^b^	6.11 ^b^	6.64 ^a^	0.12	***
Cohesiveness	0.64 ^b^	0.70 ^a^	0.65 ^ab^	0.50 ^c^	0.47 ^c^	0.02	***
Springiness (mm)	1.33 ^c^	1.04 ^d^	2.35 ^a^	1.73 ^b^	1.85 ^b^	0.07	***
Chewiness (N·mm)	3.50 ^c^	3.97 ^c^	9.03 ^a^	5.24 ^b^	5.80 ^b^	0.35	***

^1^ Level of significance: *** *p* < 0.001. ^a–d^ Different superscripts in the same row represent significant differences (*p* < 0.05). ^2^ Hue angle = tan^−1^(b*/a*). ^3^ Saturation index = (b*^2^ + a*^2^)^0.5^.

**Table 2:** Effects of searing process on cooking properties of sous-vide cooked pork patties.

	Control	Searing Treatment				SEM	Level of Significance ^1^
		S30	S60	S90	S120		
Cooking yield (%)	81.6 ^a^	80.1 ^b^	76.9 ^c^	75.4 ^d^	73.4 ^e^	0.01	***
Diameter reduction (%)	12.0 ^e^	13.1 ^d^	15.4 ^c^	18.3 ^b^	21.2 ^a^	0.26	***
Thickness increment (%)	34.5 ^b^	27.6 ^c^	23.0 ^d^	32.2 ^b^	41.1 ^a^	0.77	***
Shrinkage (%)	4.71 ^e^	6.67 ^d^	9.25 ^c^	10.2 ^b^	11.3 ^a^	0.25	***

^1^ Level of significance: *** *p* < 0.001. ^a–e^ Different superscripts in the same row represent significant differences (*p* < 0.05).

**Table 3:** Effects of searing process on visual attributes of sous-vide cooked pork patties.

	Control	Searing Treatment				SEM	Level of Significance ^1^
		S30	S60	S90	S120		
Color ^2^	3.35 ^d^	4.70 ^c^	6.35 ^b^	7.17 ^a^	7.17 ^a^	0.28	***
Moisture	6.11 ^a^	5.70 ^ab^	5.46 ^b^	4.88 ^c^	4.29 ^d^	0.22	***
Appearance	4.29 ^d^	5.41 ^c^	7.35 ^a^	6.70 ^b^	5.41 ^c^	0.26	***
Overall acceptability	4.37 ^d^	5.42 ^c^	7.54 ^a^	6.95 ^b^	5.78 ^c^	0.23	***

^1^ Level of significance: *** *p* < 0.001. ^a–d^ Different superscripts in the same row represent significant differences (*p* < 0.05). ^2^ Score distributions (1–9): color (very pale–very dark), moisture (very dry–very moisture), appearance acceptability (very unacceptable–vey acceptable), and overall acceptability (very unacceptable–very acceptable).

**Table 4:** Effects of searing process on sensory quality characteristics of sous-vide cooked patties.

	Control	Searing Treatment				SEM	Level of Significance ^1^
		S30	S60	S90	S120		
Tenderness ^2^	5.36 ^d^	6.30 ^bc^	7.24 ^a^	6.48 ^b^	5.65 ^cd^	0.29	***
Juiciness	5.73 ^a^	5.44 ^a^	5.03 ^ab^	4.55 ^bc^	3.85 ^c^	0.32	***
Flavor intensity	5.46 ^c^	5.93 ^c^	6.69 ^b^	7.16 ^ab^	7.28 ^a^	0.23	***
Off-flavor intensity	5.16	5.45	5.74	5.57	5.63	0.33	NS
Saltiness	5.73 ^a^	5.44 ^a^	5.03 ^ab^	4.55 ^bc^	3.85 ^c^	0.32	***
Overall acceptability	5.32 ^d^	6.14 ^bc^	7.14 ^a^	6.38 ^b^	5.62 ^cd^	0.27	***

^1^ Level of significance: NS = not significant; *** *p* < 0.001. ^a–^^d^ Different superscripts in the same row represent significant differences (*p* < 0.05). ^2^ Score distributions (1–9): tenderness acceptability (very unacceptable–very acceptable), juiciness (extremely dry–extremely juicy), flavor intensity (no patty flavor–full flavor), off-flavor intensity (very strong–very weak), saltiness (very weak–very strong), and overall acceptability (very unacceptable–very acceptable).

**Table 5:** Effects of searing process on total aerobic bacteria and coliforms of pork patties during storage.

	Control	Searing Treatment				SEM	Level of Significance ^1^
		S30	S60	S90	S120		
*Total aerobic plate count (log CFU/g)*							
After searing	8.43 ^a^	1.88 ^b^	1.40 ^bc^	1.07 ^c^	0.51 ^c^	0.27	***
After sous-vide treatment							
0 d	ND	ND	ND	ND	ND	ND	
14 d	ND	ND	ND	ND	ND	ND	
49 d	ND	ND	ND	ND	ND	ND	
*Coliform count (log CFU/g)*							
After searing	0.76 ^a^	0.83 ^a^	0.37 ^b^	0.10 ^c^	0.07 ^c^	0.06	***
After sous-vide treatment							
0 d	ND	ND	ND	ND	ND	ND	
14 d	ND	ND	ND	ND	ND	ND	
49 d	ND	ND	ND	ND	ND	ND	

^1^ Level of significance: *** *p* < 0.001. ^a–c^ Different superscripts in the same row represent significant differences (*p* < 0.05). ND, not detected.
